# Supplementary material for: Fabrication of Gold Nanostructures on Quartz Crystal Microbalance Surface Using Nanoimprint Lithography for Sensing Applications
Source: Micromachines (Basel). 2022 Aug 29;13(9):1430. doi: 10.3390/mi13091430 (PMC9501340; doi:10.3390/mi13091430)
Supplement: Supplementary file 1 [file micromachines-13-01430-s001.zip › micromachines-1864767-supplementary.pdf]

Supplementary Materials

Fabrication of gold nanostructures on quartz crystal microbalance surface using nanoimprint lithography for sensing applications

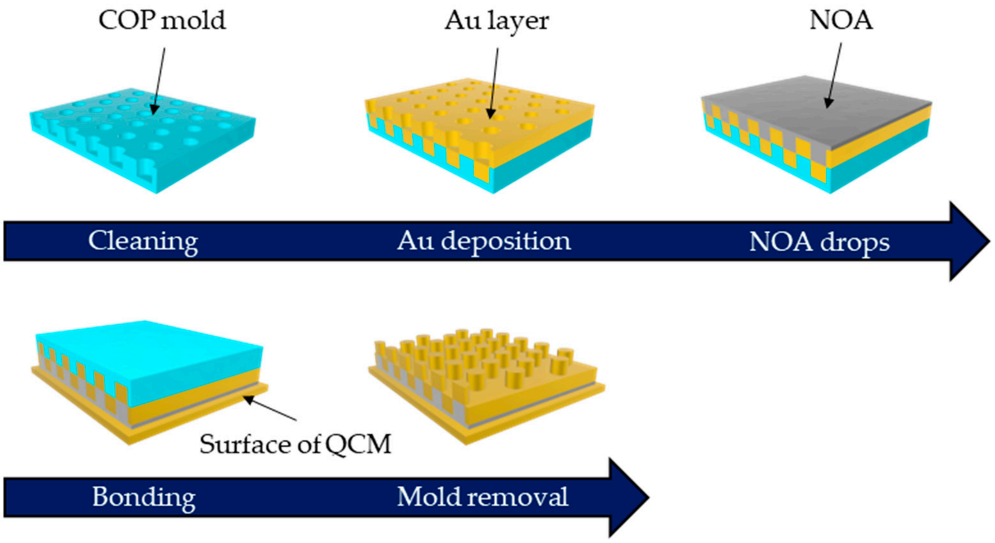

Figure S1. Schematic of the gold nanostructure fabrication.

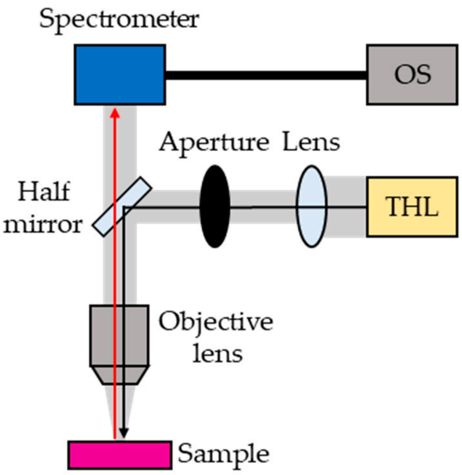

Figure S2. Schematic of the homemade optical setup for measuring the reflection spectrum.

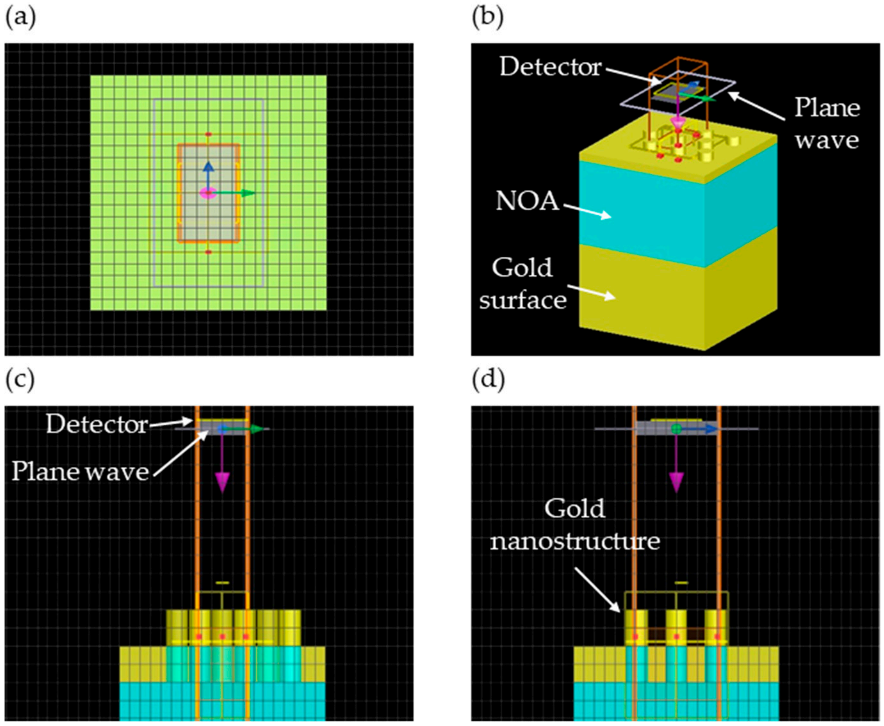

Figure S3. Details of gold nanostructure simulation model. (a) 3D view. (b) X-Y plane. (c) X-Z plane. (d) Y-Z plane.

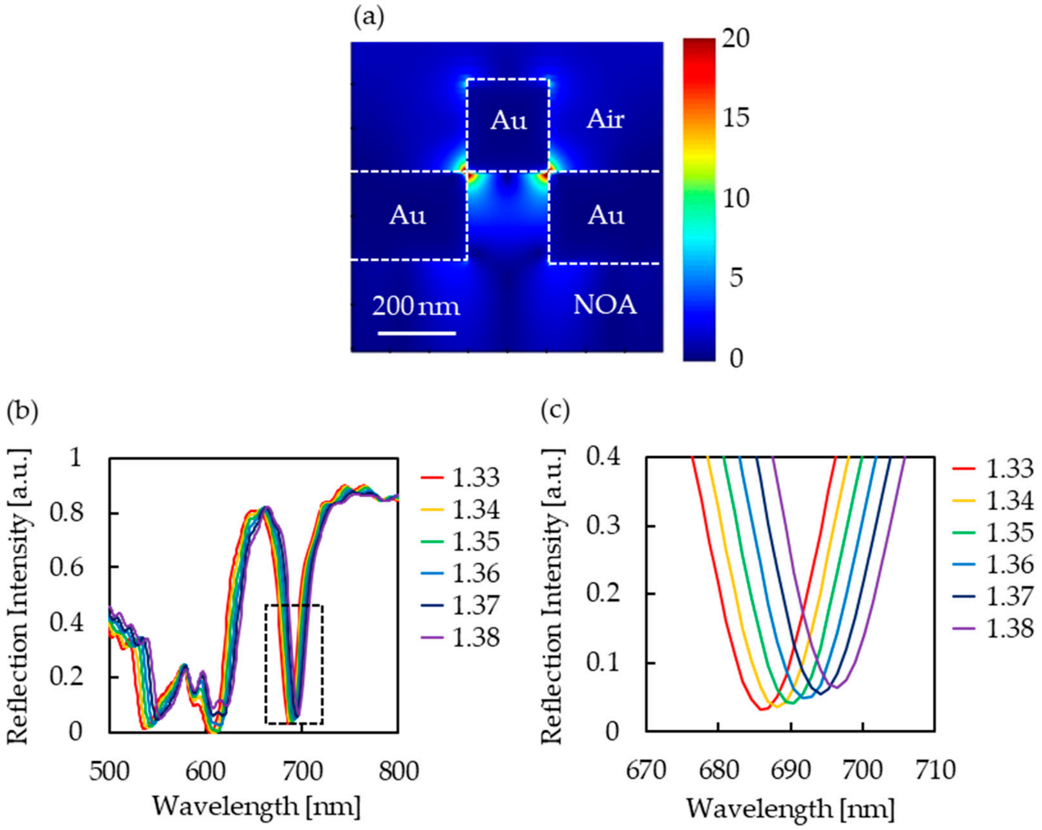

Figure S4. (a) Electric field distribution at 649 nm. (b) Reflection spectra calculated by simulation in various refractive index environments. (c) Enlarged image of the absorption peak in (b) (dotted squares).

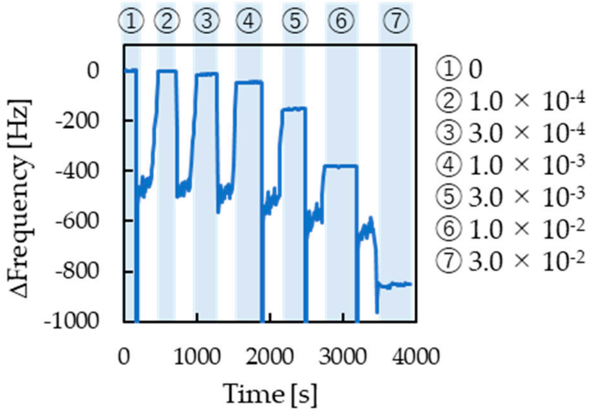

Figure S5. Resonance frequency change measured by a QCM device without nanostructure when nanoparticles (1.0×10^{-4}–3.0×10^{-2}% (w/w)) are adsorbed.
